# Exogenous interleukin-10 alleviates allergic inflammation but inhibits local interleukin-10 expression in a mouse allergic rhinitis model

**DOI:** 10.1186/1471-2172-15-9

**Published:** 2014-02-25

**Authors:** Shui-Bin Wang, Yu-Qin Deng, Jie Ren, Bo-Kui Xiao, Zheng Liu, Ze-Zhang Tao

**Affiliations:** 1 Department of Otolaryngology-Head and Neck Surgery, Renmin Hospital of Wuhan University, Wuhan, China; 2 Department of Otolaryngology-Head and Neck Surgery, Tongji Hospital, Tongji Medical College, Huazhong University of Science and Technology, Wuhan, China; 3 Department of Otolaryngology-Head and Neck Surgery, Hubei University of Science and Technology, Xianning, China

**Keywords:** Allergic rhinitis, Interleukin-10, Eosinophils, Mast cells, T-cell subsets, Regulatory T cells

## Abstract

**Background:**

Interleukin-10 (IL-10) has an important anti-inflammatory and immunoregulatory function, and its expression is negatively correlated with the development and severity of allergic rhinitis (AR). However, the in vivo effects of exogenous IL-10 on AR have not been studied and the mechanisms underlying the effects of IL-10 have not been fully understood. Here, we investigated the effects of intranasal administration of recombinant mouse (rm) IL-10 on the expression of Th responses and local IL-10 in a mouse model of AR induced by ovalbumin.

**Results:**

Administration of rmIL-10 during challenge significantly reduced the number of eosinophils and mast cells, as well as Type 2 helper T (Th2) and Th17 cell related cytokine and transcription factor levels in the nasal mucosa and nasal lavage fluid in AR mice. The rmIL-10 treatment significantly inhibited the number of IL-10-positive cells and IL-10 mRNA expression in the nasal mucosa in AR mice.

**Conclusion:**

Our results show that exogenous IL-10 administrated in challenge phase alleviates nasal allergic inflammation in AR mice, most likely by inhibiting Th2 and Th17 responses. It can also inhibit local IL-10 levels in the nasal mucosa. Our findings indicate that IL-10 may have the potential as an inhibitor of AR.

## Background

Allergic rhinitis (AR) is one of the most prevalent airway diseases worldwide, which exerts a heavy burden on patients due to its impact on physical, social and emotional functioning, as well as the cost of treatment. Despite the increasing worldwide prevalence of AR, treatments for AR remain limited in effect [1]. Thus, it is greatly important to develop novel therapies with higher efficacy for AR patients.

AR is characterized by a disturbance of T-cell subsets and an accumulation of eosinophils and mast cells. CD4^+^ T-cell subsets are central regulators of immune responses and allergic inflammatory diseases. After encountering specific antigen, they become activated, proliferate, and differentiate into specific Type 1 helper T (Th1), Th2, Th17, or regulatory T (Treg) cells distinguished by cytokine profile, surface phenotype, and specific transcription factor expression. AR has long been considered as an allergic response involving predominantly Th2 cells, with a relative insufficiency of Th1 cells [2]. However, in recent years, the classic paradigm of Th1/Th2 cell-mediated immunity has been evolved to include novel subsets of Th cell, such as Th17 cells and Treg cells. Exaggerated Th17 and impaired Treg responses have been implicated in the development of AR and have been associated with corticosteroid resistance [2-7]. These T-cell subsets regulate inflammatory responses mainly by secreting specific cytokines such as interferon-γ (IFN-γ), IL-5, IL-17, IL-10, and transforming growth factor-β1 (TGF-β1), as well as other non-specific cytokines such as tumor-necrosis factor-α (TNF-α) [3-6,8,9].

IL-10 is a multifunctional cytokine secreted by a variety of cells, including T cells, monocytes, macrophages, dendritic cells, and endothelial cells. IL-10 has diverse effects on most hemopoietic cells. Its crucial role is to limit and ultimately terminate immune and inflammatory responses [10]. In the sensitization phase of allergic responses, IL-10 can down-regulate major histocompatibility complex class II and costimulatory molecule expression on dendritic cells and induce anergic immune responses possibly through up-regulating cytotoxic T lymphocyte-associated molecule-4 (CTLA)-4 expression. In the effector phase, IL-10 directly inhibits active CD4^+^ T cells proliferation and their function by crosslinking IL-10R on the surface of activated CD4^+^ T cells, which engages the Jak family signalling pathway of tyrosine kinases Jak1 and Tyk2 [11]. Because of its inhibitory properties, IL-10 has been suggested as a potential therapy for systemic and localized autoimmune and allergic inflammatory diseases, including inflammatory bowel disease, allergic asthma, rheumatoid arthritis, and experimental autoimmune encephalomyelitis [10,12,13]. It has been reported that endogenous IL-10 levels in the nasal endothelium are decreased and negatively correlated with AR development and severity in AR patients [14,15], which makes us hypothesize that IL-10 may alleviate allergic inflammation in AR. Here, we investigated the effect of intranasal exogenous IL-10 administration on the allergic inflammation of AR in a mouse model induced by ovalbumin (OVA).

## Results

### rmIL-10 reduces the number of eosinophils and mast cells in nasal mucosa in AR mice

**Figure 1:**
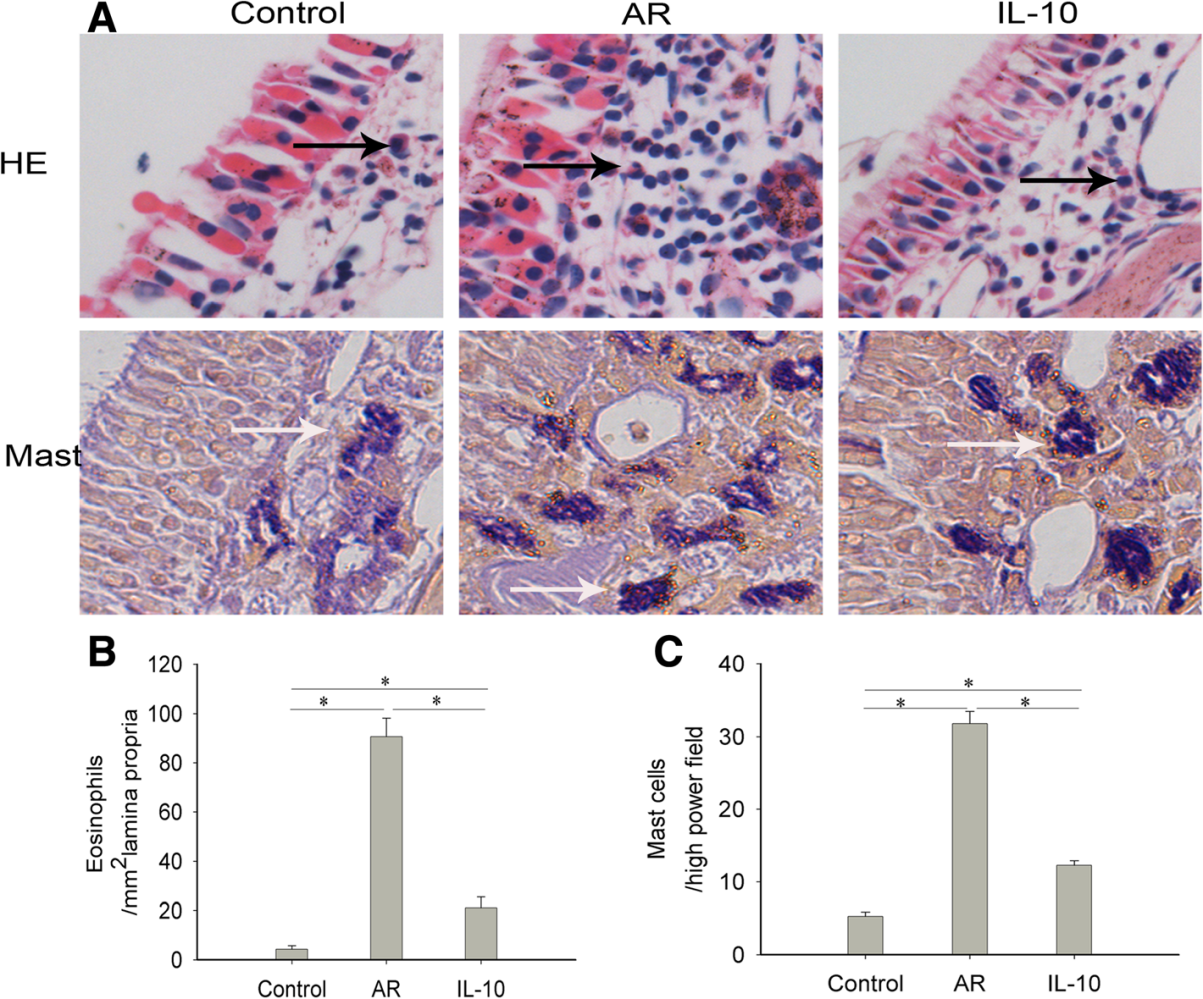
**rmIL-10 reduced the infiltration of eosinophils and mast cells in nasal mucosa.** Representative photomicrographs (original magnification × 400) of nasal mucosa sections of mice stained with haematoxylin and eosin (H&E) for eosinophils (black arrows) or by mast cell staining for mast cells (white arrows) **(A)**. A significantly increased number of eosinophils **(B)** and mast cells **(C)** in the lamina propria (LP) were observed in the AR group compared with the control group, and such increase was obviously alleviated by rmIL-10 treatment in the IL-10 group. Control group, saline-sensitized and challenged mice; AR group, ovalbumin-sensitized and challenged mice; IL-10 group, AR mice were treated with rmIL-10 during challenge.

To determine the in vivo effects of rmIL-10 on AR, an OVA-induced mouse AR model was used. The IL-10 group received 0.1 μg/mouse of rmIL-10 simultaneously with OVA challenge. The control group received PBS sensitization and challenge instead of OVA. Mice were sacrificed and nasal mucosa inflammation was evaluated at 24 hours after the final challenge (day 28). The infiltration of eosinophils and mast cells was mainly found in the lamina propria (LP) of nasal mucosa. The cytoplasm was stained red for eosinophils with H&E staining, while the cytoplasm was stained purple for mast cells with mast cell staining (Figure 1A). The number of eosinophils and mast cells was significantly increased in AR group compared with that seen in the control group (p < 0.05), and this increase was markedly inhibited by rmIL-10 treatment (p < 0.05) (Figure 1B and C).

### rmIL-10 administration increases the total IL-10 protein levels but decreases other cytokine levels in nasal lavage fluid

**Figure 2:**
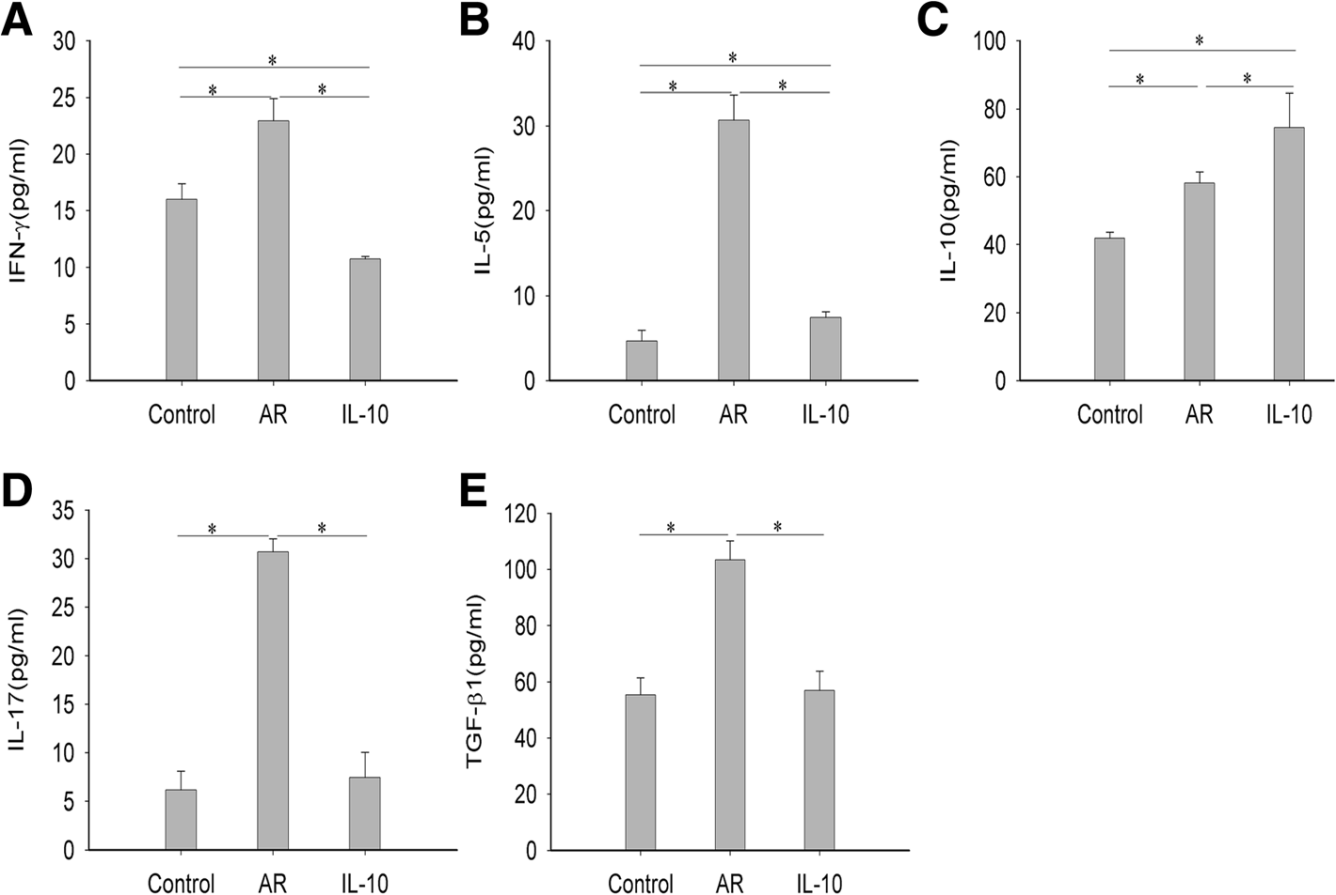
**rmIL-10 administration increased total IL-10 levels but decreased other cytokine levels in NLF.** Cytokine levels in nasal lavage fluid (NLF) were assessed by means of ELISA. Compared with the control group, the levels of all cytokines in the NLF were significantly elevated in AR group, and rmIL-10 administration during challenge markedly reduced the levels of IFN-γ, IL-5, IL-17, and TGF-β1 **(A-B and D-E)**, but further increased the IL-10 levels **(C)**. Control group, saline-sensitized and challenged mice; AR group, ovalbumin-sensitized and challenged mice; IL-10 group, AR mice were treated with rmIL-10.

To determine the effects of rmIL-10 on the Th cell related cytokine levels in the nasal cavity, the protein levels of IFN-γ, IL-5, IL-17, IL-10, and TGF-β1 in the nasal lavage fluid (NLF**)** were analyzed by ELISA. Compared with those seen in the control group, the levels of IFN-γ, IL-5, IL-17, and TGF-β1 in NLF were significantly increased in the AR group (p < 0.05), and such up-regulation was markedly inhibited by rmIL-10 treatment (p < 0.05) (Figure 2A, B, D, and E). The levels of IL-10 in NLF were also significantly increased in AR group in comparison with those seen in the control group (p < 0.05), but was further increased with rmIL-10 treatment (p < 0.05) (Figure 2C).

### rmIL-10 differentially modulates mRNA expression of Th cell related cytokines and T-cell subset transcription factors in nasal mucosa

**Figure 3:**
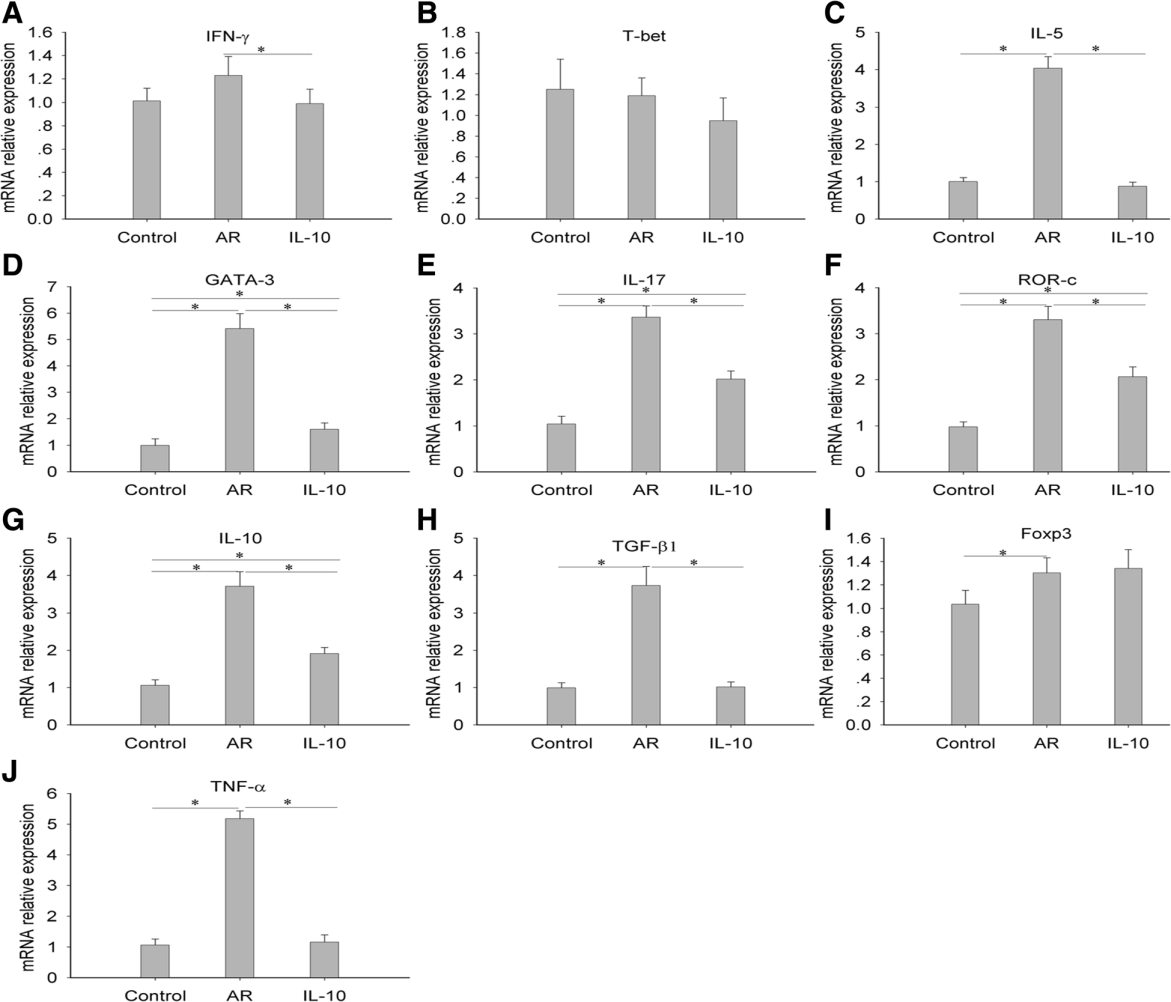
**rmIL-10 differentially modulated mRNA expression of Th subsets related cytokines and transcription factors in nasal mucosa.** The mRNA relative expression levels of IL-5, IL-17, IL-10, TGF-β1, and TNF-α **(C, E, G, H, and J)** were significantly up-regulated in the AR group compared with the control group, and such up-regulation was markedly inhibited in AR mice with rmIL-10 treatment during challenge. Compared with the control group, IFN-γ mRNA expression levels **(A)** moderately increased without statistical significance,, but were significantly down-regulated with rmIL-10 treatment in AR mice. Compare with the control group, AR mice demonstrated significantly up-regulated the mRNA expression levels of GATA-3 and ROR-c in the nasal mucosa that were markedly suppressed with rmIL-10 treatment **(D and F)**. Although AR mice had moderately increased Foxp3 mRNA expression levels compared with controls, but rmIL-10 treatment had no effect on Foxp3 mRNA expression **(I)**. There was no significant difference for T-bet mRNA expression levels among different groups **(B)**. T-bet, T-box transcription factor; GATA-3, GATA binding protein 3; ROR-c, retinoic acid-related orphan receptor C; Foxp3, Forkhead box P3. Control group, saline-sensitized and challenged mice; AR group, ovalbumin-sensitized and challenged mice; IL-10 group, AR mice were treated with rmIL-10 during challenge.

To further determine the effects of rmIL-10 on allergic airway inflammation, we used qPCR to examine the mRNA expression of Th cell related cytokines and T-cell subset transcription factors in the nasal mucosa. Significantly up-regulation of mRNA expression levels of IL-5, IL-17, IL-10, TGF-β1, and TNF-α were found in the AR group compared with that seen in the control group (p < 0.05), and this up-regulation was markedly decreased after treatment with IL-10 in the IL-10 group (p < 0.05) (Figure 3C, E, G, H, J). IFN-γ mRNA expression levels were comparable between the control and AR groups, whereas it was slightly decreased in the IL-10 group compared with the AR group (p < 0.05), (Figure 3A). The mRNA expression levels of GATA-3 and ROR-c (Figure 3D, F) were significantly higher in the AR group than in the control group and significantly lower in the IL-10 group than in the AR group (p < 0.05). Foxp3 mRNA expression levels were slightly increased in the AR group compared with those seen in the control group (p < 0.05), whereas rmIL-10 had no significant effect on its expression (Figure 3I). T-bet mRNA expression levels were comparable across different study groups (Figure 3B).

### rmIL-10 reduced local IL-10–positive cells in nasal mucosa

**Figure 4:**
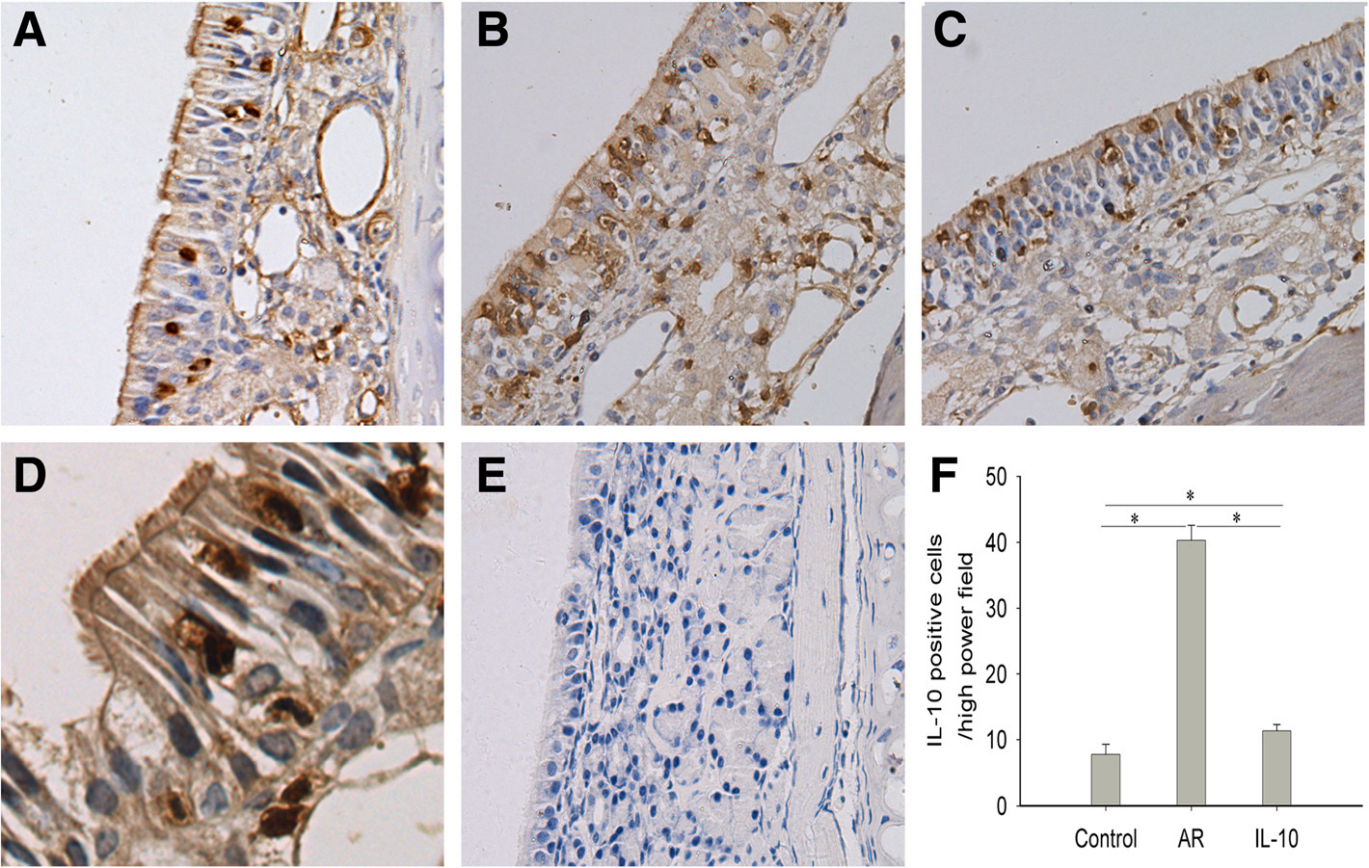
**rmIL-10 reduced local IL-10 expression in nasal mucosa.** IL-10 immunohistochemistry for IL-10-positive cells in the nasal mucosa of mice was carried out by using the streptavidin-biotin complex method. Representative photomicrographs (original magnification × 400) of IL-10 immunohistochemical staining of nasal mucosa sections from the control mice **(A)**, AR mice **(B)**, and AR mice treated with rmIL-10 **(C)**, and sections stained with negative control for anti-IL-10 antibody **(E)**. A representative photomicrograph with enlarged magnification (original magnification × 1000) of IL-10 immunohistochemical staining of nasal mucosa sections from AR mice **(D)**. The number of IL-10-positive cells was significantly higher in the AR group compared with the control group, and it was reduced in the AR group compared with the IL-10 group **(F)**. Control group, saline-sensitized and challenged mice; AR group, OVA-sensitized and challenged mice; IL-10 group, AR mice were treated with rmIL-10 during challenge.

The protein expression and cellular location of IL-10 in the nasal mucosa were investigated using immunohistochemistry. IL-10 immunoreactivity was found in epithelial cells and infiltrating inflammatory cells in LP (Figure 4A-D). No immunoreactivity was observed in nasal mucosa stained with the negative control (isotype rabbit IgG) for anti-IL-10 (Figure 4E). The number of IL-10-positive cells in the nasal mucosa was significantly increased in the AR group compared with that seen in the control group (p < 0.05), and such increase was markedly suppressed with IL-10 treatment in the IL-10 group (p < 0.05), (Figure 4F).

## Discussion

In this study, we demonstrated that rmIL-10 administration during challenge could alleviate allergic inflammatory response in AR mice, which was evidenced by the decreased infiltration of eosinophils and mast cells, and reduced Th2 and Th17 responses. In contrast, interestingly, rmIL-10 inhibited the local IL-10 expression in the nasal mucosa.

AR is characterized by the accumulation of eosinophils and mast cells. Th cells and their secreted cytokines play a central role in regulating the migration, apoptosis, and function of eosinophils and mast cells in AR. Th2 cells secrete IL- 4, IL-5, IL-10, and IL-13, which mount humoral immunity by inducing IgE production by B cells and by activating mast cells and eosinophils, while Th1 cells mediate cellular immunity by secreting IFN-γ. It has been considered that the function of Th1 and Th2 cells is inhibited by each other [2]. In line with a previous finding, we found that T-bet expression was not significantly changed in AR mice compared with the controls [16]. However, IFN-γ expression levels in the NLF and nasal mucosa were slightly increased in AR mice compared with the controls, and were down-regulated with rmIL-10 treatment during challenge. The inconsistent expression between IFN-γ and T-bet was not very surprising. It has been reported that IFN-γ is secreted by a variety of cells, including Th1 cells, macrophages, and epithelial cells, but T-bet is only expressed by Th1 cells. We also showed that Th2 responses (IL-5, IL-10, and GATA-3 expression) were markedly down-regulated with rmIL-10 treatment, suggesting that IL-10 can suppress Th2 reactions [12,13].

IL-17-producing Th17 cells, induced in vitro by IL-6, IL-23, and TGF-β1, and regulated by transcription factor ROR-c, have multiple functions in different inflammatory airway diseases. Th17 cells have not only been indicated in control of the mucosal pathogen, but also are involved in the pathogenesis of Th1-mediated inflammatory diseases [3,4]. Recently, Th17 cells have been associated with Th2 predominated allergic diseases. Increased expression of Th17 responses has been found in asthma, AR, and nasal polyps [5,17-19]. Th17 cells have been demonstrated to promote the development and augment the severity of allergic inflammatory airway diseases by secreting pro-inflammatory cytokines and chemokines, such as IL-1β, IL-6, IL-8, and IL-17 [5,17,18]. Interestingly, increased IL-17 expression is associated with resistance to corticosteroid in AR patients [20]. Consistent with previous reports [5,7], we showed that the expression levels of IL-17, ROR-c, and TGF-β1 were significantly increased in AR mice compared with the control mice. Moreover, we found that the increased expression of Th17 responses could be significantly down-regulated by IL-10 treatment in AR mice. A probable underlying mechanism may be that IL-10 combines IL-10R on the surface of activated Th17 cells and inhibits proliferation and function of Th17 cells [11]. It has also been reported that an intermediate subset of T cells, Foxp3^+^ IL-17^+^ T cells, exist in the nasal mucosa of patients with AR, which can be converted to Foxp3^+^ Treg under the influence of IL-10 [21]. In this study, we found an inhibition of ROR-c expression without an increase of Foxp3^+^ expression. Therefore, we could not preclude the possibility that rmIL-10 may down-regulate Th17 responses by promoting the conversion of Foxp3^+^ IL-17^+^ T cells to Foxp3^+^ Treg [21]. However, this needs further investigation in future.

Unlike other effector CD4^+^ T cells, Treg cells have been implicated in peripheral tolerance as an inhibitor of immune responses. Treg cells are classified into natural Treg (nTreg) generated in the thymus and induced Treg (iTreg) cells induced by IL-10 or TGF-β1 in the periphery. Both of them are required for the full expression of tolerance but have a division of labour in immune homeostasis. nTreg cells migrate preferentially to lymphoid organs, prevent the development of autoimmune disease, and can be recruited and activated early during an immune response to control its magnitude, whereas iTreg cells are induced upon repeated antigen stimulation, act later to reduce the activation of T cells directed against environmental antigens and to restore and maintain tolerance [22-25]. One of the iTreg cells is Type 1 Regulatory T (Tr1) cells that are induced by IL-10. A few studies have demonstrated that iTreg cells also express Foxp3 upon activation. However, Foxp3 expression on Tr1 cells is low and transient or never reaches the high expression levels that characterize Foxp3^+^ Treg. Furthermore, Foxp3 is not required for Tr1 cell induction or function since suppressive Tr1 cells can be generated or isolated from peripheral blood of patients with Foxp3-mutations, even in those patients with complete deletion of Foxp3 [26,27]. Therefore, Tr1 cells may not express Foxp3 and their immunosuppressive effects are partly inhibited by anti-IL-10 neutralizing antibody [27]. Tr1 cells, but not nTreg cells, were decreased in the peripheral blood of patients with AR [25,28]. In the present study, total Foxp3 mRNA expression was slightly increased in AR mice compared with the controls, reflecting a possible increased recruitment of nTreg to nasal mucosa or activation of T cells in nasal mucosa. IL-10 treatment may induce accumulation of Tr1 in nasal mucosa, however, the limited amount of murine nasal mucosa does not allow the flow cytometric analysis of Tr1 in nasal mucosa based on the co-expression of CD49b and lymphocyte activation gene 3 on Tr1 [26].

We found that IL-10 protein levels in NLF, and IL-10 mRNA expression level and IL-10-positive cell number in the nasal mucosa were significantly increased in AR mice compared with the control mice. Immunohistochemistry showed that IL-10-immunoreactivity was mainly in epithelial cells and inflammatory cells in LP. Previous studies have demonstrated that IL-10-positive cells are mainly T and B lymphocytes, monocytes, macrophages, and mast cells in LP in allergic responses [10,29]. In this study, we found that rmIL-10 treatment further increased IL-10 protein levels in NLF. In order to determine if increased IL-10 protein levels in NLF came from the exogenous rmIL-10 or induced local IL-10 expression in nasal mucosa, we investigated the IL-10 mRNA and protein expression in the nasal mucosa and found that IL-10 expression in nasal mucosa was markedly decreased with rmIL-10 treatment. The results indicate that exogenous IL-10 treatment can inhibit the local IL-10 expression in the nasal mucosa and the increased IL-10 protein levels in NLF are derived from exogenous rmIL-10.

In the study, local treatment with rmIL-10 significantly alleviated Th2 and Th17 responses in the nasal mucosa. The increased expression of IL-17 has been shown to be negatively associated with the sensitivity of AR patients to topical corticosteroid treatment [20]. IL-10 can upregulate glucocorticoid-receptor expression of asthmatic CD4^+^ T cells with glucocorticoid-resistant [29]. Thus, IL-10 may have potential benefits for the treatment of AR patients with corticosteroid resistance. It is well accepted that Th1 and Treg cells play an important role in constraining allergic reactions. Their under-expression or dysfunction is closely related to the development of AR. Our study suggests that IL-10 administration at current dose may not cause significant suppression to the immunity against allergic airway responses since it had not apparently inhibitory effect on the expression of Th1 and Treg cells.

Although glucocorticoids have been used for the treatment of inflammatory and allergic airway diseases for a long time, their side effects have been concerned for many years, such as growth inhibition, osteoporosis, and gastric ulcer [30-32]. Based on its anti-inflammation function, intranasally delivered IL-10 has the potential to be an alternative drug for glucocorticoids and therefore to avoid side effects of glucocorticoids.

Our current study has several limitations. One is that given the limited amount of mouse nasal tissues, we could not perform the flow cytometric analysis of Th subsets at cellular level. Second, our AR mouse model was established by OVA sensitization and challenge. It may be better to induce AR with relevant allergens found in human AR, such as pollens and dust mites. Third, we administrated exogenous IL-10 during challenge instead of after the establishment of AR. Therefore, the therapeutic effect of IL-10 on established AR needs to be studied in the future research.

## Conclusions

In conclusion, the study demonstrates that rmIL-10 administration during challenge down-regulates allergic reactions in the context of AR, most likely through inhibiting Th2 and Th17 responses without impairment of Th1 and Treg responses. Exogenous IL-10 can also inhibit local IL-10 expression in the nasal mucosa in the context of AR. Given its anti-inflammatory and immunosuppressive functions, IL-10 may have a promising future as an inhibitor of AR. However, this needs further studies in future.

## Methods

### Animals

C57BL/6 J mice (6–8 weeks old) were purchased from the Shanghai Experimental Animal Center (Shanghai, China). These animals were kept in a special pathogen-free biohazard containment facility and were used following protocols approved by the Animal Care and Use Committee of Renmin Hospital of Wuhan University.

### AR murine model establishment and rmIL-10 administration

A total of 48 mice were randomly divided into the control, AR, and IL-10 groups with 8 mice in each group, and 2 independent experiments were performed. OVA (Grade V, Sigma, St. Louis, MO) was used for allergen sensitization and challenge to establish the AR mouse model according to the method previously described [16]. Briefly, on days 0, 7, and 14, mice were immunized by intraperitoneal injection of 25 μg OVA and 1 mg aluminium hydroxide in 300 μl phosphate-buffered saline (PBS), and followed by daily intranasal challenge (from day 21 to 27) with 2.5% OVA in 40 μl PBS (20 μl for each nose). The control group received PBS sensitization and challenge instead of OVA. The IL-I0 group received 0.1 μg rmIL-10 (PeproTech, USA) administration simultaneously with OVA challenge continuously for 7 days [12].

### Nasal lavage

Four mice per group were underwent nasal lavage before histopathology. Nasal lavage was carried out using the method previously described with slight modifications [33]. The nasal cavity was gently irrigated with l ml of sterile saline, and the nasal lavage fluid (NLF) was collected and centrifuged. The supernatants were stored at −20°C for cytokine measurement using enzyme-linked immunosorbent assay (ELISA).

### Measurement of cytokines in NLF

The levels of IL-5, IL-10, IL-17, TGF-β1, and IFN- γ in NLF were measured by means of ELISA. The ELISA kits were all from Boster Biotech (Wuhan, China) and their detection sensitivity was 2 pg/ml for all the cytokines. All the procedures were performed according to the manufacturer’s instructions.

### Histopathology

Four mice per group were underwent histopathology. Sections were prepared as described elsewhere [34]. Histological changes in the nasal mucosa were examined through haematoxylin and eosin (HE) staining for eosinophils and mast cell staining for mast cells. Eosinophils and mast cells in the LP were counted in 4 randomly selected fields. The results for eosinophils and mast cells were expressed as numbers /mm^2^ and numbers per high-power (HP) field, respectively.

### RNA extraction and quantitative real-time RT-PCR

Four mice were selected from each group and the nasal mucosa were obtained and homogenized as described elsewhere [34]. Total RNA was extracted by Trizol (Invitrogen, Carlsbad, CA, USA) and 0.5 μg of total RNA was used for the reverse-transcription reaction using a Rever Tra Ace qPCR RT Kit (TOYOBO, Japan) according to the manufacturer’s instructions. The qPCR was carried out for the detection of mRNA expression of T-bet (NM_019507.2), GATA-3 (NM_008091.3), ROR-c (NM_011281.2), Foxp3 (NM_001199348.1), IFN-γ (NM_008337.3), IL-5 (NM_010558.1), IL-17 (NM_010552.3), IL-10 (NM_010548.2), TGF-β1 (NM_011577.1), and TNF-α (NM_013693.2). The reaction was performed with an ABI 7500 Real-time PCR system (Applied Biosystems, USA) using the SYBR qPCR Mix (TOYOBO, Japan) according to the manufacturer’s protocol. An analysis of relative expression was calculated using the 2^-ΔΔCT^ method as described elsewhere, and one sample from a control served as a calibrator [35]. Glyceraldehyde-3-phosphate dehydrogenase (GAPDH, NM_008084.2) was used as a housekeeping gene for normalization, and a no-template sample was used as a negative control.

### Immunohistochemistry

Rabbit anti-mouse polyclonal anti-IL-10 antibody (Boster Biotech, Wuhan, China) was diluted by 1:100. The immunoreactivity was detected using a Streptavidin-Biotin Complex (SABC) Kit (Boster Biotech, Wuhan, China) and 3, 3′-diaminobenzidine tetrahydrochloride, which rendered positive cells brown. Control isotype rabbit IgG was used as a negative control [35]. The number of IL-10-positive cells per high-power field of epithelium and LP was counted.

### Statistical analysis

All results are expressed as the means ± SEM. Results from the different groups were compared using the non-parametric Kruskal-Wallis test, and followed by the Mann–Whitney U test. Statistical analysis was performed using IBM SPSS Statistics 19.0 software (IBM, USA), and p < 0.05 was considered statistically significant.

## Abbreviations

AR: Allergic rhinitis; HE: Hematoxylin-eosin; OVA: Ovalbumin; HP: High power; LP: Lamina propria; NLF: Nasal lavage fluid; ELISA: Enzyme-linked immunosorbent assay; rm: Recombinant murine; IL: Interleukin; Th: Helper T; IFN-γ: Interferon-gamma; TGF-β1: Transforming growth factor-beta1; TNF-α: Tumor necrosis factor-alpha; T-bet: T-box transcription factor; GATA-3: GATA binding protein 3; ROR-c: Retinoic acid–related orphan receptor C; Foxp3: Forkhead box P3; Tregs: Regulatory T cells; GAPDH: Glyceraldehydes −3 phosphate dehydrogenase.

## Competing interests

The authors declare that they have no competing interests.

## Authors’ contributions

SBW performed experimental studies and drafted the manuscript. YQD and BKX performed the main animal experiments. JR analyzed data and performed the statistical analysis. ZL prepared and revised the manuscript. ZZT participated in its design and prepared the manuscript. All authors have read and approved the final manuscript.
